# High-Quality Draft Genome Sequence of the Xanthomonas translucens pv. cerealis Pathotype Strain CFBP 2541

**DOI:** 10.1128/genomeA.01574-14

**Published:** 2015-02-12

**Authors:** Céline Pesce, Stéphanie Bolot, Sébastien Cunnac, Perrine Portier, Marion Fischer-Le Saux, Marie-Agnès Jacques, Lionel Gagnevin, Matthieu Arlat, Laurent D. Noël, Sébastien Carrère, Claude Bragard, Ralf Koebnik

**Affiliations:** a UMR 186 IRD-Cirad-Université Montpellier 2 Résistance des Plantes aux Bioagresseurs, Montpellier, France; b Earth and Life Institute, Applied Microbiology Phytopathology, Université Catholique de Louvain, Louvain-la-Neuve, Belgium; c INRA, Laboratoire des Interactions Plantes Micro-organismes (LIPM), UMR 441, Castanet-Tolosan, France; d CNRS, LIPM, UMR 2594, Castanet-Tolosan, France; e INRA, UMR1345 Institut de Recherche en Horticulture et Semences (IRHS), Angers, France; f Agrocampus Ouest, UMR1345 IRHS, Angers, France; g Université d’Angers, UMR1345 IRHS, SFR4207 QUASAV, PRES L’UNAM, Angers, France; h INRA, CIRM-CFBP Collection Française de Bactéries associées aux Plantes, Angers, France; i UMR Peuplements Végétaux et Bioagresseurs en Milieu Tropical (PVBMT), CIRAD, Saint-Pierre, La Réunion, France; j Université Paul Sabatier, Toulouse, France

## Abstract

Xanthomonas translucens pv. cerealis is the causal agent of bacterial leaf streak on true grasses. The genome of the pathotype strain CFBP 2541 was sequenced in order to decipher mechanisms that provoke disease and to elucidate the role of transcription activator-like (TAL) type III effectors in pathogenicity.

## GENOME ANNOUNCEMENT

Wheat and other small grain cereals are major crops worldwide and are considered important 4F (food, feed, fiber, and fuel) plants. In human consumption, wheat ranks as the second most-produced crop plant after rice, and wheat is grown on more land area than any other commercial crop (see http://faostat3.fao.org/home/E).

Xanthomonas translucens pv. cerealis has been found on crops, like wheat (Triticum spp.), barley (Hordeum spp.), and rye (Secale cereale) (1–3), and it also naturally occurs on smooth bromegrass and quack grass (4). Bacterial leaf streak caused by strains of X. translucens (5) is the most common bacterial disease of wheat. As a seed-borne disease, it is a constraint for international germplasm exchange (6). The symptoms include translucent stripes at the leaf blade at the early infection state, which later develop into elongated water-soaked lesions, as well as the production of exudates at late infection state (7). While most plant-pathogenic xanthomonads studied thus far belong to the group II clade, the strains of X. translucens belong to the group I clade, which also includes the species Xanthomonas albilineans, Xanthomonas hyacinthi, Xanthomonas sacchari, and Xanthomonas theicola (8).

Pathotype strain CFBP 2541 (LMG 679, NCPPB 1944) was isolated from Bromus inermis in the United States in 1941. We tested this strain on barley (Hordeum vulgare L. Morex and Betzes) and wheat (Triticum aestivum L. Alondra) under laboratory conditions. Strong symptoms were obtained with the “Morex” and “Alondra” plants, while “Betzes” remained symptomless.

To obtain new insights into the molecular determinants provoking disease or resistance, we sequenced strain CFBP 2541 using the Illumina HiSeq 2000 platform (GATC Biotech, Germany). The shotgun sequencing yielded 59,447,151 read pairs (26,337,209 100-bp paired-end reads, with an insert size of 250 bp, and 33,109,942 50-bp mate-pair reads, with an insert size of 3 kb). A combination of Velvet (9), SOAP*denovo*, and SOAPGapCloser (10) yielded 31 contigs >500 bp (*N*_50_, 1,399,657 bp), with the largest contig being 1,809 kb, for a total assembly size of 4,515,938 bp, corresponding to 1,926× coverage.

The genome was found to encode a noncanonical hypersensitive response and pathogenicity (Hrp) type III protein secretion system, the genetic organization of which differs from that of clade II xanthomonads, as previously reported for X. translucens pv. graminis strain Xtg29 (11). In contrast to strain Xtg29, however, the genome assembly of strain CFBP 2541 indicated the presence of two type III transcription activator-like (TAL) effector genes (12, 13), which was supported by Southern blot hybridization. Since *tal* genes are notoriously difficult to be assembled from short reads due to their repetitive nature, we sequenced the *tal* genes upon PCR amplification. Surprisingly, the two genome-assembled *tal* genes turned out to be correctly assembled, probably due to the very high coverage and a significant number of single-nucleotide polymorphisms (on average, 1 per 10 bp) that distinguish all individual repeats from each other. This information opens the way for studying the role of *tal* genes in the pathogenicity of X. translucens.

### Nucleotide sequence accession numbers.

This whole-genome shotgun project has been deposited at DDBJ/EMBL/GenBank under the accession no. JWHD00000000. The version described in this paper is the first version, JWHD01000000.
